# Approaches to manage ‘affordability’ of high budget impact medicines in key EU countries

**DOI:** 10.1080/20016689.2018.1478539

**Published:** 2018-06-08

**Authors:** Mathias Flume, Marc Bardou, Stefano Capri, Oriol Sola-Morales, David Cunningham, Lars-Ake Levin, Maarten J. Postma, Nicolas Touchot

**Affiliations:** a Kassenärztliche Vereinigung Westfalen-Lippe (KVWL), Dortmund, Germany; b CIC INSERM 1432, CHU CHU Dijon-Bourgogne, Dijon Cedex, France; c School of Economics and Management, Cattaneo-LIUC University, Castellanza (Varese), Italy; d Health innovation technology Transfer, Barcelona, Spain; e Cunningham Healthcare Advisory, Croydon, Surrey, UK; f Department of Medical and Health Sciences, Linköping University, Linköping, Sweden; g Department of Health Sciences, University of Groningen, University Medical Center Groningen, Groningen, The Netherlands; h groupH, London, UK

**Keywords:** Budget impact, affordability, cost effectiveness, market access, funding, expensive therapies, health technology assessment, price negotiation

## Abstract

**Background:** The launch of hepatitis C (HCV) drugs such as sofosbuvir or ledipasvir has fostered the question of affordability of novel high budget impact therapies even in countries with high domestic product. European countries have developed a variety of mechanisms to improve affordability of such therapies, including ‘affordability thresholds’, price volume agreements or caps on individual product sales, and special budgets for innovative drugs. While some of these mechanisms may help limit budget impact, there are still significant progresses to be made in the definition and implementation of approaches to ensure affordability, especially in health systems where the growth potential in drug spending and/or in the patient contribution to health insurance are limited. **Objectives:** In this article, we will review how seven countries in western Europe are approaching the question of affordability of novel therapies and are developing approaches to continue to reward new sciences while limiting budget impact. We will also discuss the question of affordability of cost-effective but hugely expensive therapies and the implications for payers and for the pharmaceutical industry. **Results: **There is clearly not one solution that is used consistently across countries but rather a number of ‘tools’ that are combined differently in each country. This illustrates the difficulty of managing affordability within different legal frameworks and within different health care system architectures.

## Introduction

Target 8E of the United Nations Millennium Development Goals acknowledges the need to improve the availability of affordable medicines in developing countries [1] and significant, albeit insufficient progresses have been made in that regard. However, following the launch of highly expensive drugs targeting very large populations such as hepatitis C drugs or PCSK9 inhibitors (proprotein convertase subtilsin-kexin type 9 – latest class of cholesterol-lowering drugs), the question of affordability of new medicines has also now emerged in developed countries.

In the USA, the high price of prescription drugs was a topic for policy proposals from presidential candidates [2], and the need to balance affordability with incentives for innovation was recently discussed in the New England Journal of Medicine [3]. In Europe, most countries define yearly, and sometime mid-term, growth targets in drug spending. Those could range from virtually no growth such as in England to a few percent per year as in Germany. Meeting these targets is increasingly challenging and drug reimbursement has to be balanced with the mandate to provide high quality care to all without discrimination on the basis of age, income or other socio-economic considerations. Furthermore, increase in drug spending competes with funding of physicians, hospitals, nurses and other health or social expenses.

Gilead’s hepatitis C Sovaldi (sofosbuvir) illustrates the challenges in achieving that balance. Despite a cure rate greater than 95% and despite the therapy being accepted as cost-effective [4], several European health systems were forced to limit budget impact through prescription delays, limits on target patients or caps on revenues. The World Health Organization recently presented a detailed analysis of budget impact of treating patients with hepatitis C with sofosbuvir or ledipasvir/sofosbuvir [5]. Treating just 20% of hepatitis C patients would consume about 20% of annual drug expenditure in Italy and Spain, 6% in England, and 4% in Germany and France.

Affordability of novel innovative and high budget impact therapies has become an important topic in Europe and so far, each country has come up with individual approaches to improve affordability. While some may be efficacious, at least partially, there is no consistent mechanism and this is creating an increasingly complex environment for companies introducing innovative medicines. The objective of this review is to describe how seven countries in western Europe are approaching the question of affordability of novel therapies, and are developing approaches to continue to reward new sciences while limiting budget impact. We will also discuss the question of affordability of cost-effective but hugely expensive therapies and the implications for payers and for the pharmaceutical industry.

## Material and methods

The information described in this paper comes from the personal experience of the authors who are payers, ex-payers or researchers in the field of Market Access in their respective countries. This knowledge was complemented by targeted secondary research on the question of affordability of novel therapies. For the analysis we chose to cover the five main western Europe countries. We also included Sweden and The Netherlands as the subject of ‘affordability’ is being extensively discussed in the payer communities in these two countries.

## Germany

In Germany, the legal framework has established a formal review process (Arzneimittelmarkt- Neuordnungsgesetz – AMNOG) to determine the extent of patient relevant additional benefit for novel therapies [6] followed by a formal national negotiation process. In that process, G-BA (Gemeinsamer Bundesausschuss or Federal Joint Committee) does not evaluate the appropriateness of the price, does not look at cost-effectiveness and does not consider the overall budget impact. In the subsequent price negotiation between manufacturer and GKV-Spitzenverband (Head organization of the Statutory Health Insurance Funds), the budget impact is taken into account, but as there is no fixed drug budget, affordability is not a formal factor in the price negotiation.

Affordability is more relevant in the hospital setting. Drugs for inpatient use have to be purchased by the hospitals out of their diagnosis related (DRG) reimbursement. If they do not receive additional funding over DRG reimbursement, high cost drugs can be a challenge.

There is no ongoing discussion about a general threshold to guarantee affordability of drugs. Only the sales volume in the initial 12-month period of free pricing with full reimbursement was discussed to be limited. But this suggestion did not make it into the AMVSG (Arzneimittelversorgungsstärkungsgesetz) law in 2017 and is currently not on the political agenda [7].

Affordability in Germany thus must be reflected within the yearly prescription spend negotiations between the Association of Physicians and the Association of Sick Funds. Specific bodies are advising physicians on the use of novel expensive drugs in order to remain within prescription limits. Recommendations are usually not developed before the G-BA issues its verdict. After the price negotiation, physicians are sometimes informed which patients really benefit and need an immediate treatment. Sick funds also try to enter into additional agreements with manufacturers about discounts. But their negotiation power is limited as they cannot prevent physicians from prescribing an expensive drug with an additional benefit.

There is an ongoing discussion at political level as to whether the current pricing policies of Pharma companies are sustainable for Germany, especially as companies may introduce drugs with additional benefits at very high prices. The experience until now shows that politicians in Germany are not willing to deny funding for drugs with an additional benefit. Even the practical rejection of some new drugs with no additional benefit by limiting them to the price of a cheap generic Standard of Care is under discussion. Politicians currently prefer to achieve savings by freezing prices [7] or asking for mandatory discounts for all branded products rather than by rejecting coverage of specific products based on an affordability criteria.

## France

In France, until very recently the budget impact of novel therapies was not a formal factor in the evaluation, coverage decision or pricing process. The focus was on avoiding off-label use through a clear definition of the target patient population by Haute Authorité de Santé (HAS), followed by price volume agreements reducing the incentive for the industry to increase sales over a certain threshold.

However, the need to manage budget impact is increasingly becoming a concern and has led to several discussions between payers and the pharmaceutical industry. Since 2016, products subject to economic evaluation by HAS (Incremental Therapeutic Progress – ASMR III requested, >€20million in sales expected) must also submit a budget impact estimate if sales during the second year of commercialization are above €50million [8]. Between €20million and €50million, the budget impact estimate is not mandatory but recommended. The estimate should not simply define the target population but also the size of the likely treated population.

At present the professed objective is not to limit access to drugs over a certain budget impact threshold, but rather to anticipate budget needs and the growth of pharmaceutical expenditures. However, it is clear that the pricing committee (CEPS) will take into account the expected budget impact, even though it will remain informal and no budget impact threshold will be defined. While nominal prices are often high, real prices can be much lower with discounts and price volume agreements renegotiated on a regular basis.

To provide funding for innovative drugs France is trying to generate economies in other areas of health spending [9]. Over the last years a number of products with minimal clinical value have been de-reimbursed [10]. The use of generics which is low compared to other European countries has been increased and additional savings should come from growing use of ambulatory care. In the future, high budget impact drugs will be covered partially through these saving, partially though increases in drug budgets and partially through additional price volume agreements.

## England

Recent amendments to the National Institute for Health and Care Excellence (NICE) assessment process were launched in April 201 [11]. These were agreed with the National Health Service (NHS) England and include three key elements. Firstly, a proposal to fast track access to technologies offering exceptional value for money (likely cost per extra year of quality-adjusted life of under £10,000). Secondly, the introduction of a budget impact test or ‘affordability criteria’ of twenty million pounds (£20million) per year as an improved way to manage treatments that are cost-effective, but have a very high cost. Thirdly, a sliding, but increased, cost per extra year of quality-adjusted life up to £300,000 per quality-adjusted life year (QALY).

For products that are likely to fall over the £20million threshold, companies will have the opportunity to enter in confidential negotiations with NHS England, to help avoid and minimize delays in patients having access to treatments recommended by NICE [12]. Should this not be possible and an agreement to minimize the impact of those drugs cannot be reached, NHS England will be able to choose to apply to NICE for an extended period in which to introduce the drug in a phased way. This will usually be for no more than three years. The phased implementation would most likely follow the one undertaken for HCV, where initial access is for patient groups with the highest clinical benefit or unmet need.

The proposed affordability criteria is a way to ensure a smooth and affordable access rather than no access. There is no appetite, both politically or managerially within the NHS, to significantly amend the current NICE cost-effective methodology. Previous efforts to move to a value-based approach were not a success and given other more pressing priorities for all parties it is likely that at least for the short term there will be no significant changes to the current value-based reimbursement system. But, cost-effectiveness on its own, especially during times of reducing healthcare expenditure or where any increase in expenditure is less than increase in demand, has been shown to be insufficient and has been demonstrated to put a strain on both access to new and innovative products, not just pharmaceuticals, but also healthcare budgets. The success of the proposed ‘budget impact threshold’ will be evaluated in the coming 3–5 years and will impact the need for additional changes in coverage policies.

## Italy

The recent Italian Medicines Agency (AIFA) algorithm for the evaluation of novel medicines does not include affordability [13]. It does not even include cost-effectiveness, to the dismay of several Italian health economists [13]. As a result, affordability plays no role in coverage decisions and the power of regions in managing drug expenses has even been reduced for drugs with a high level of innovativeness that are now automatically and immediately included on regional formularies.

Italy relies on three main tools to reduce budget impact of novel therapies: price negotiations, cap on specific drug expenditures and performance-based schemes. While the definition of a ‘cap’ is a quite effective way to limit budget impact, it is negotiated for individual products based on expected target population and is in fact more a tool to avoid off-label use. Of interest is the recent evolution in performance-based schemes. Those were traditionally based on a refund from the industry to the health system upon failure of achieving efficacy metrics. However, refunds proved to be minimal and incomplete [14]. Risk-sharing agreements may now also include a ‘success fees’ (example pirfenidone for Idiopathic Pulmonary Fibrosis), whereby the National Health System makes the payment to the manufacturer only for patients who received a real benefit, meaning that payment follows provision of value [14]. This is perceived by Italian payers as a way to limit the immediate impact on health budgets and to be protected from uncertainties on efficacy.

To help afford expensive innovative drugs, Italy has also created a specific funding mechanism, putting aside €500million for oncology drugs and another €500million for non-oncology drugs^18^. Initially non-oncology meant only drugs for HCV but other drugs (e.g. Strimvelis) have been added and will be added in the future [15]. Should spending reach over €500million, for example due to an increase in the number of patient treated in the indications covered by that special fund, the industry will have to pay back the spending above €500million, leading to a lower net cost per patient. So this special budget could be in some way considered as an affordability threshold, but not for individual drugs, rather by groups of product (highly innovative oncology and non-oncology).

Finally, negotiation appears to be the strongest tool in order to achieve affordability, as shown by the Sovaldi example. Through the combination of price negotiations, caps and paybacks, the price of Sovaldi has dramatically diminished from an initial €45,000 ex-factory price to a real net price of about €15,000 per treatment. The pay-back from Gilead to AIFA from July 2015 until December 2016 has been of €935million [16]. Unfortunately, this is only a special case, confirming that there is yet not transparent and standard mechanism to manage affordability of high budget impact drugs in Italy.

## Spain

The approach from the Spanish authorities to affordability has not significantly changed since the 2008 financial crisis when several legislations were passed forcing an unnegotiated price reduction to some of the drugs already in the market, plus important compulsory discounts for drugs provided to hospitals [17]. Regions reacted to the latter by shifting some products to be delivered from hospitals instead of community pharmacies, and some nursing home supply to be directly managed from hospital [18].

The Sovaldi case opened the door to budget caps per drug set at the national level [19]. This has been implemented for several drugs, but more recently this option has lost traction in the verge of the difficult allocation of benefits across the regions. At present, affordability is still very much transferred to the regions, and managed through budgetary control. By transferring budgets to hospitals and limiting their ability to grow, regions impose priority setting on the hospitals, which have to be balanced with waiting lists.

The idea of centralised purchasing has been gaining momentum; the national government has indeed organised some centralised tenders for some devices [20], and many regions have initiated centralised purchasing or at least price negotiation [21]. One clear trend has been to centralise some regional decision making in the access to high cost drugs, either by selecting (explicitly or implicitly) reference centres, or by directly requiring pre-authorisation of high cost treatments (i.e. oncology) to a central regional commission.

However, to keep within budgets and ensure sustainability, most of the onus remains on price negotiation at the provider level and some access restrictions at the regional level. On the price side, there has been a progressive de-link between the national price level (list price) and the reimbursed price. Price re-negotiations have become more and more common at regional and hospital level, and simple discounts being sought. Some regions have started imposing flat-fees per disease (DRG type of payment), whilst annually reducing the amount in line with expected discounts. Performance based agreements have been signed as in many other countries, but to a limited extent, as many hospitals or institutions have found the transaction costs to high.

Interestingly, the national Government and the pharma trade representatives keep signing maximum increases on the level of expenditure, although implementation of such agreements seem not to take place.

## Sweden

When making decisions the Swedish reimbursement authority (The Dental and Pharmaceutical Benefits Agency – TLV) has to follow three principles that are included in the Health and Medical Service Act: (i) the human-dignity principle, (ii) the needs-solidarity principle and (iii) the cost-effectiveness principle [22]. When making the decisions there is a need to balance those principles which are often in conflict with each other. One way to solve the quite obvious conflict between the principle of needs-solidarity (which means that more health care resources should be allocated to patients with greater needs and worse quality of life) and the principle of cost-effectiveness is through balancing the cost per QALY threshold, allowing higher cost-effectiveness thresholds (ICER) for treatments of more severe conditions [23].

The legislation does not mention affordability as principle, nor is there a debate on affordability thresholds. However, the health care system has to handle affordability issues when they appear. The first example of this, within the current reimbursement system was the introduction of the new hepatitis-C during 2014. The problem was that the drugs were found to be cost-effective but their funding was associated to a perceived risk of undesired displacement effects in other areas of the health care system due to very high budget impact.

After concluding that the health care did not have the capacity in the short term to treat all patients who could receive treatment (regardless of severity), TLV weighed into their decision that priority should be given to those with the greatest need [24]. TLV decided to subsidize these drugs with the restriction that they were only reimbursed for patients in fibrosis stages F3 and F4. For treating patients with lower stages and therefore with less severe disease, the drugs were not reimbursed. So in practice TLV used the principle of need and solidarity to restrict the reimbursement of new drugs that were potentially not affordable. The risk and the magnitude of the undesired displacement effects are however still unknown.

## Netherlands

In the Netherlands, there is no maximum affordability threshold. Budget impact is used to define the need for detailed assessment [25]: if it is predicted below EUR 2.5 million on the national level, no extensive assessment is done. Above that level, the Ministry of Health stratifies new technologies by low, medium and high risk innovations due to (i) the height of the initial budget impact, (ii) the potential increase of the budget impact in the years after market launch, (iii) the price of the drug (>EUR 50,000/year is considered as a red flag) and (iv) the ICER and the methodology of the cost-effectiveness model. The ICER and the model’s methodology are assessed by ‘Zorginstituut Nederland’. If any of the above aspects are deemed as ‘high risk’, the pharmaceutical company is invited by the Ministry of Health to start, generally on the price, until the budget impact and ICER are acceptable. There is an informal maximum cost-effectiveness threshold at EUR 80,000/QALY for ‘end-of-life drugs’). There is no (in)formal affordability threshold in this later stage of the assessment.

To improve affordability the Dutch health system relies on traditional tools of price negotiations, define starting and stopping rules and price/volume agreements. Additionally, the Dutch Health Council may provide some informal guidance on prescribing in their advisory reports (this was, for example, the case with the new oral anti-coagulants and hepatitis C drugs). More recently joint price negotiations with Belgium, Luxemburg and Austria have been used to enhance negotiating power [26]. However, at present, if the cost effectiveness model is valid and transparent, and if the ICER threshold is met, a drug cannot formally be rejected on the basis of affordability or because of its impact on the prescription budget [25]. There is no current discussion on establishing an affordability threshold for budget impact, current political discussions focus on mechanisms to lower drug prices that are within the realm of the Ministry of Health its power. For example, a re-definition of the Law on Drugs’ Prices (‘Wet Geneesmiddel Prijzen’).

## Comparison between countries

The analysis of seven European countries shows a great variability in approaching or handling the affordability question (Table 1).

**Table 1.** Approaches to ensure affordability of novel high budget impact therapies.

Country	Affordability Threshold	Cap on Volume or Price Volume Agreement	Restriction on population	Special funding for expensive drugs	Limit in pharma expenditure increase/patient contributions	Informal Guidance to Prescription
Germany		√				√√√
France		√√	√		√√	
England	√√	√√	√√	√√	√	√
Italy		√√	√	√√√	√	
Spain		√√	√		√√	√
Sweden			√√√			
Netherlands		√√√	√			√

There is clearly not one solution that is used consistently across countries but rather a number of ‘tools’ that are combined differently in each country. This illustrates the difficulty of managing affordability within different legal frameworks and within different health care system architectures.

## Discussion

While it is clear that novel approaches and mechanisms are needed few countries have implemented formal ‘affordability thresholds’. The only one with a meaningful formal threshold is actually England even though caps on volume or price volume agreements informally play a similar role in several other EU countries. The question of affordability may become even more acute if NASH (Nonalcoholic steatohepatitis) develops to a multi-billion-dollar indication as forecasted by the financial industry [27], if Disease Modifying Therapies for Alzheimer’s Disease reach market, or when expensive one-off ‘curative’ therapies enter the market for large indications such as Gene Therapies for Haemophilia or for hypercholesterolemia. As several novel high budget impact therapies come to market, replicating the budget impact of hepatitis-C therapies multiple times over, it is likely that most EU countries will have to increase the use of approaches to increase affordability. However, we still do not expect to see a homogeneous pan European approach, but more a combination of approaches that will be adapted to each country health system and to diverse ‘cultural’ attitudes toward restrictions of therapy coverage.

In these discussions one stakeholder group that so far has played little impact is politicians, except in England for cancer therapies. One interesting example comes from Ireland where a debate has been going on for several years between The Department of Health and Health Service Executive (HSE) for funding on novel high budget impact drug. It started with the funding of Kalydeco® for Cystic Fibrosis. The drug was initially considered not cost-effective but following an intensive lobbying campaign by patients and families, the Ministry of Health stepped in to ensure funding by HSE [28]. The debate is now replicated for a group of nine high-tech drugs for which HSE claims not to have funding. The Ministry of Health had initially agreed to review funding requests for high-tech drugs outside of HSE budget but has now changed its position saying that such funding should come from savings within HSE [29]. This illustrates the difficulty politicians have with tackling the question of affordability of new high budget impact therapies. In most other countries, politicians have been careful to avoid significant involvement in this question.

For high budget impact therapies, the use of European price reference is losing relevance. In England, confidential discounts may be first necessary to meet NICE cost per QALY requirements, and then additional confidential discounts may be required to overcome the £20million affordability threshold. In France, the price volume agreement leads to net prices that are widely different from negotiated ‘face’ price. Similarly, in other countries real net prices per patient are often far lower than official prices even when those are negotiated at national level. As a result, many of the past efforts to ensure price transparency have been rendered null. This goes against the demands of payers in most EU countries and will either lead to informal exchange of confidential information or to higher variability in price across countries, neither of which is a desired outcome.

Another important question emerging for payers is the relationship between affordability and cost-effectiveness. Not all cost-effective therapies are affordable, as illustrated in England by the £20million affordability threshold that also applies to drugs deemed cost-effective by NICE, and by TLV in Sweden by its decision to limit the new hepatitis-C drugs to a specific patient population. NASH is again a good example of the likely payer dilemma. Health economic models developed by the industry are likely to support use of the novel therapies. But savings will be realized in the long-term, often out of the payer budget cycle and after spending of large sums of money on the indication for many years. So how can payers protect themselves against the uncertainty when long-term clinical benefits and savings turn out to be lower than expected? Of course, payers will likely become even more stringent about cost-effectiveness models and uncertain assumptions to avoid situations such as hepatitis-C drugs where long-term data may not confirm initial analysis. But raising the bar even higher could lead to denial of coverage of very useful therapies. Italy provides one interesting alternative: initial low payment to increase short-term affordability followed by subsequent additional payment if certain criteria representing value for patient (not surrogate) are met. But while interesting this approach may be difficult to implement in countries such as Germany where patients often change health insurance and may not be attractive to the pharma industry as it could significantly delay revenues.

Paying for performance is often mentioned as a way to cope with affordability allowing a faster penetration of innovations to the market. However, while this can be an effective tool in some cases, this is not always true. Paying for performance contracts are regularly used by the industry during price negotiations to maintain or increase official list/reimbursed prices. From a company point of view this is usually a better approach than agreeing to a lower official price, especially in countries that are used internationally for reference pricing. But the net effect on affordability is often limited compared to the lower official negotiated price that would have been required without the pay for performance contract.

One option that has not gained enough attention is financial based agreements, by which drugs/new technologies would be delivered for an agreed fee for a specific population, irrespective of the actual number of patients treated. Such an approach has been discussed for antibiotics and would allow payers to better anticipate spending in a given therapeutic area/patient population, ensuring affordability, or at least leading to enhanced affordability decisions.

The ‘affordability question’ is also very confusing for the industry. Revenue modelling for an innovative high budget impact drug requires specific construct for each major EU country. Not only effective price will vary country per country, but so will covered patient population and not entirely on the basis of clinical efficacy or cost-effectiveness but to a high degree simply on the basis of affordability. This is a criterion that is most difficult to forecast and depends upon external factors such as overall economic situation and potentially launch in completely unrelated therapeutic areas. While the industry has been good to forecast the impact of competitive launch within the same indication, it has little experience forecasting the impact of the launch of a high budget impact product in totally unrelated indications. Pharma companies will need to move from ‘competitive intelligence’ to a broader ‘budget impact intelligence’ to account for future affordability issues.
